# Comparison of association between physical activity and resting metabolic rate in young and middle-aged Korean adults

**DOI:** 10.20463/jenb.2019.0012

**Published:** 2019-06-30

**Authors:** Hyejung Hwang, Won-Sang Jung, Jisu Kim, Hun-Young Park, Kiwon Lim

**Affiliations:** 1 Physical Activity and Performance Institute (PAPI), Konkuk Universit, Seoul Republic of Korea; 2 Department of Physical Education, Konkuk University, Seoul Republic of Korea

**Keywords:** physical activity, body composition, resting metabolic rate, aging

## Abstract

**[Purpose]:**

The purpose of this study was to comparatively investigate the correlation among body composition, resting metabolic rate (RMR), and physical activity (PA) between young and middle-aged Korean adults.

**[Methods]:**

A total of 53 [male n=23, female n=30] subjects were included in this study, among whom 34 subjects were healthy young adults [male n=18, female n=16] and 19 were middle-aged adults [male n=5, female n=14]. The body composition and RMR of all the participants were measured after overnight fasting (≥8 h). The Korean version of the WHO Global Physical Activity Questionnaire (GPAQ) was used to assess physical activity.

**[Results]:**

Body composition was not significantly different between young adults and middle-aged adults. Whole-body bone mineral density and bone mineral contents (BMC) were significantly lower in middle-aged adults than in young adults. Total blood cholesterol (TC) and blood glucose levels were significantly higher in middle-aged adults (TC; 195.21 ± 43.34, glucose; 103.57 ± 12.61 mg/dL) than in young adults. RMR was significantly lower in middle-aged adults (1619.57 ± 290.28 kcal/day) than in young adults (1894.37 ± 405.00 kcal/day). In middle-aged adults physical activity (PA). PA (METs, min, EE) was inversely correlated with fat mass (FM, kg, and %) and blood triglyceride (TG) level in young adults. In middle-aged adults, PA showed a significant positive correlation with lean body mass (LBM), FM (%), and RMR. Furthermore, PA EE showed significant interrelatedness with BMC among middle-aged adults.

**[Conclusion]:**

These results demonstrated that high PA levels enable LBM and RMR maintenance in middle-aged adults. Furthermore, in young adults, more PA is required to induce change in body composition.

## INTRODUCTION

Physical inactivity is a direct risk factor for obesity, metabolic syndrome, cancer, and depression^1-4^. These diseases are threats to an individual’s physical and economic wellbeing. Currently, the main reason for the decline in physical activity (PA) is sedentary lifestyle, leading to obesity^5^. Obesity, which has increased in numbers over the last few decades, is one of the most serious health concerns. A Korean adult is deemed to be obese if the body mass index (BMI) is 25 (kg/m^2^) or more. Over the past decade, obesity rates in Korean men and women have increased by 4.9% and 1.5%, respectively^6^. The prevalence of obesity varies with age and changes in body composition and resting metabolic rate (RMR), caused by aging^7^. Therefore, it is essential to understand the relationship between age and PA requirement, and the importance of maintaining an active lifestyle. Many studies were performed on the correlation among PA, body composition, and energy metabolism^8-11^. However, earlier studies did not demonstrate change in age-related body composition, PA, and RMR because subjects of different ages were not compared. Particularly, an analysis of the relationship between Koreans’ age and body composition, RMR, and PA has not been undertaken yet. Therefore, this study was designed to comparatively investigate the correlation among body composition, RMR, and PA between young and middle-aged adults.

## METHODS

### Participants and experiments design

**Figure 1.**
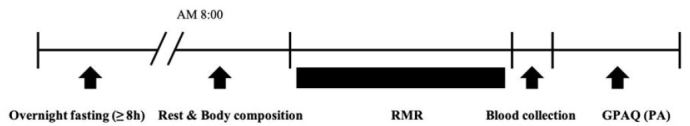
Schematic representation of the experimental design.

Thirty-four healthy young adults [mean± standard deviation (SD) age: 23.63 ± 2.20 years, male n=18, female n=16] and 19 middle-aged adults [47.36 ± 5.63 years, male n=5, female n=14] were included in the present study. Criteria for study subjects’ recruitment were as follows: no history of orthopedic and medical diseases for the past 1 year and participation in the PAR-Q & YOU (PA preparation questionnaire) and the modified AHA/ACSM health questionnaire. We selected 53 participants who reported no health problems in the pre-screening surveys and had received doctor's clearance for participation in the study. We excluded those with thyroid disease, type 1 and type 2 diabetes, cardiovascular disease, severe hypertension, more than 6% weight loss during the past six months, and pregnant women and those under extensive aerobic and aerobic exercise training. All participants were explained about the experiment purpose, procedures, and the potential risks of the present study. All proceedings of the study were approved by the Institutional Review Board of Konkuk University (7001355-201903-HR-305) in Korea and were conducted according to the Declaration of Helsinki. Written consent to participate in the study was obtained from each participant. The experimental design is presented in Fig. 1. All participants arrived at the laboratory early in the morning (8:00 AM) after overnight fasting (≥8 h) and rested for thirty minutes, after which their body composition was measured, followed by RMR measurement. After measuring body composition and RMR, blood samples were collected. The participants completed the Global Physical Activity Questionnaire (GPAQ) on the same day.

### Body composition and blood sample assessment

Body height, weight, and whole-body composition (lean body mass; LBM, Fat mass; FM) were measured by electrical bioimpedance analysis methods using body composition analyzer (InBody 770, Biospace Ltd, Seoul, Korea). The participants wore regular indoor clothing and were instructed to stand barefooted in an upright position on the machine platform, with their feet on the electrodes in the platform, while their hands gripped the wires on the handles. Bone mineral content was measured by dual-energy X-ray absorptiometry (DEXA) methods using whole-body DXA bone densitometer (PRIMUS, OsteoSys, Seoul, South Korea). Participants wore regular clothing without metals to minimize clothing absorption. Blood samples were collected after measuring body composition. Total cholesterol (TC), triglyceride (TG) level, and blood glucose levels were measured using a portable digital lipid analyzer (SD LipidoCare, SD Biosensor, Inc., Seoul, Korea), and fresh capillary whole blood samples were collected from each participant by fingerstick.

### RMR

Each participant underwent an overnight fast (≥8 h), after which RMR was measured by indirect calorimetry using a metabolic gas analyzer (Quark CPET, Cosmed, Rome, Italy) with a flow-dilution canopy hood system to determine VO_2_, carbon dioxide output, and the respiratory exchange ratio. Calibration was performed using calibration gas (16% O_2_ and 5% CO_2_) before the measurement. The measurement room had controlled conditions of humidity (50%) and temperature (23 ± 1 °C). All participants were asked to limit their PA and abstain from alcohol intake the day before the measurement. Before the measurement, the participant took a rest of 30 minutes, and RMR was measured on the supine position for 30 minutes. The presented mean RMR for each participant is on the 25 minute from the last measurement data.

### Physical activities

To estimate the PA level, we used the Korean version of the WHO GPAQ. We asked the participant to recollect the activities routinely performed in a week, and the total amount of PA was calculated by referring to the method of calculating GPAQ score^12^. PA level as metabolic equivalent (MET) per week and time (min) per week and sedentary time (min) per day were calculated according to GPAQ analysis guidelines. Energy consumption during PA per week was calculated using the MET Equation [1 MET = 3.5 mL O_2_/kg/min].

### Statistical analysis

All data were expressed as mean ± SD and statistical analyses were performed using IBM SPSS Statistics 22 (SPSS Inc., Chicago, IL, USA). Comparison of variables by age and sex was done using independent-samples t-test. The correlations among body composition, RMR, and PA were determined using Pearson correlation coefficient (r). We used Cohen’s d effect size, in which the term effect size refers to the statistical value calculated from a data sample and standardized mean differences. A significance level of < 0.05 was used to determine statistical difference for the mean of effect size, and the confidence interval was set at 95 %.

## RESULTS

Table 1 shows the body composition, blood variables, and the RMR of participants. Body composition (body mass index: BMI, lean body mass: LBM, fat mass: FM [kg and %]) did not present a significant difference between young adults and middle-aged adults. However, whole-body bone mineral density (BMD) and bone mineral contents (BMCs) were significantly lower in middle-aged adults (1.10 ± 0.10 g/cm^2^, 2222.43 ± 364.33 g) than in young adults (1.23 ± 0.92 g/cm^2^, 2811.40 ± 515.96 g, *p*<0.01**). TC and blood glucose levels were significantly higher in middle-aged adults (TC; 195.21 ± 43.34, glucose; 103.57 ± 12.61 mg/dL, *p*<0.05*) than in young adults. However, blood TG levels tended to be higher in young adults, although all the blood values were within the normal range, than in middle-age adults. Moreover, RMR was significantly lower in middle-aged adults (1619.57 ± 290.28 kcal/day, *p*<0.05*) than in young adults (1894.37 ± 405.00 kcal/day). These results indicated a typical age difference^13,14^. 

**Table 1.** General characteristics of the subjects

Variables	Young adults (age range 20-27 years)			Middle-aged adults (age range 40-55 years)			Cohen’s *d*
	Male (n=18)	Female (n=16)	Total (n=34)	Male (n=5)	Female (n=14)	Total (n=19)	
Age(yeas)	24.27 ± 2.49	22.93 ± 1.52	23.63 ± 2.20	47.00 ± 5.43	47.50 ± 5.91	47.36 ± 5.63	-5.074
Height(cm)	177.43 ± 7.77	159.47 ± 4.30	168.48 ± 10.83	173.60 ± 3.27	161.77 ± 5.18	164.83 ± 7.10	0.472
Weight(kg)	75.37 ± 9.97	53.87 ± 6.10	64.93 ± 13.74	76.00 ± 6.88	55.72 ± 4.88	61.05 ± 10.57	0.333
BMI(kg/m^2^)	23.86 ± 2.03	21.19 ± 2.28	22.62 ± 2.55	25.20 ± 1.26	21.29 ± 1.34	22.31 ± 2.28	0.120
LBM(kg)	61.11 ± 8.14	37.16 ± 2.84	49.21 ± 13.29	60.48 ± 4.36	39.49 ± 3.96	45.01 ± 10.28	0.420
Fat mass(kg)	14.2 ± 5.29	16.70 ± 4.42	15.71 ± 4.73	14.82 ± 2.12	16.06 ± 4.11	15.73 ± 3.67	-0.071
Fat mass(%)	14.26 ± 5.29	30.01 ± 6.06	24.56 ±7.67	19.42 ± 1.74	28.64 ± 6.28	26.21 ± 6.82	-0.283
BMD(g/cm^2^)	1.27 ± 0.07	1.16 ± 0.05	1.23 ± 0.92	1.10 ± 0.10	1.11 ± 0.11	1.10 ± 0.10**	1.260**
BMC(g)	3236.31 ± 399.99	2382.98 ± 127.14	2811.40 ± 515.96	2630.88 ± 193.17	2076.55 ± 291.94	2222.43 ± 364.33**	1.434**
TC(mg/dL)	176.50 ± 26.73	170.50 ± 28.46	173.67 ± 26.52	215.40 ± 25.22	188.00 ± 46.83	195.21 ± 43.34*	-0.640*
TG(mg/dL)	136.53 ± 78.46	161.06 ± 72.71	148.33 ± 68.49	135.00 ± 47.72	133.67 ± 65.36	134.00 ± 59.90	0.208
Glucose(mg/dL)	94.55 ± 8.55	95.43 ± 11.55	94.97 ± 10.16	112.40 ± 19.48	100.43 ± 7.93	103.57 ± 12.61*	-0.690*
RMR(kcal/day)	2210.01 ± 281.26	1558.99 ± 180.71	1894.37 ± 405.00	2010.99 ± 264.55	1479.78 ± 123.16	1619.57 ± 290.28*	0.849*

Values are means and standard deviation (mean ± SD), * *P* < 0.05 between groups and ** *P* < 0.01 between groups.

BMD: bone mineral density, BMC: bone mineral contents, TC: blood total cholesterol, TG: blood total triglyceride, RMR: resting metabolic rate

Table 2 presents the level of total PA in young and middle-aged adults. There was no significant difference in a week’s total PA (METs), the sum of time performing PA (time), and total energy expenditure of PA (EE) between young adults and middle-aged adults, even though the middle-aged group tended to show higher average PA.

**Table 2.** Comparison of total physical activity (moderate-to-vigorous activity during work, leisure, and transport time)

Physical Activity	METs (week)	Time (min/week)	Energy Expenditure (EE, kcal/week)
Young adults	2033.54 ± 1790.83	435.80 ± 367.61	2371.41 ± 2299.13
Middle-aged adults	2347.13 ± 1434.65	546.78 ± 332.20	3083.36 ± 1990.73

Values are means and standard deviation (mean ± SD),

* *P* < 0.05 between groups

Table 3 shows the correlation analysis among body composition parameters, RMR, and PA in young adults and middle-aged adults. A significant correlation coefficient was observed at the *p*<0.05 and *p*<0.001 levels. Particularly, PA (METs, min, EE) had inverse correlations with FM (kg and %), and blood TG level in young adults. In middle-aged adults, PA had a significant positive correlation with LBM, FM (%), and RMR. Furthermore, PA EE also showed significant interrelatedness with BMC among middle-aged adults (r =.456, *p*<0.05*).

**Table 3.** Pearson correlations analyses of body composition, resting metabolic rate and physical activity

Young adults											
	BW (kg)	BMI (kg/m^2^)	LBM (kg)	Body fat (kg)	Body fat (%)	BMC (g)	BMD (g/cm2)	TG (mg/dL)	TC (mg/dL)	Glucose (mg/dL)	RMR (kcal/day)
PA (METs/week)	0.046	-0.072	0.24	-0.529**	-0.477**	0.17	0.002	-.440*	-0.11	0.068	0.005
PA (min/week)	-0.026	-0.121	0.141	-0.455**	-0.372*	0.086	-0.072	-.468**	-0.162	0.102	-0.055
PA (kcal/week)	0.23	0.076	0.413*	-0.497**	-0.556**	0.338	0.15	-.406*	-0.111	0.047	0.176
Middle-aged adults											
PA (METs/week)	0.422	0.287	0.557*	-0.373	-0.590**	0.42	0.099	-0.307	-0.082	0.073	0.534*
PA (min/week)	0.436	0.305	0.561*	-0.34	-0.569*	0.416	0.1	-0.323	-0.062	0.106	0.547*
PA (kcal/week)	0.492*	0.355	0.585**	-0.26	-0.523*	0.456*	0.145	-0.23	-0.113	0.09	0.555*

*Significant correlation *P* < 0.05. BW: body weight, BMI: body mass index, LBM: lean body mass, BMD: bone mineral density, BMC: bone mineral contents, TC: blood total cholesterol, TG: blood total triglyceride, RMR: resting metabolic rate

## DISCUSSION

The major findings of the present study are as follows: firstly, body composition (LBM, FM, and BMI) showed no significant difference between young adults and middle-aged adults. However, BMD and BMC were significantly lower among middle-aged adults. Blood variables (TC, glucose) were significantly higher and RMR was significantly lower in middle-aged adults. Secondly, the interrelatedness among PA (METs, time, EE), FM, and blood TG concentration was significant in young adults, while PA and FM percentage (%) had significant negative correlations. Meanwhile, PA showed significant positive correlations with LBM and RMR among middle-aged adults.

It is well known that aging is associated with decreased LBM, and increased FM in the body, thereby reducing the RMR^7,13^. Moreover, age is associated with increased blood parameters (TG, TC, glucose), an indicator of health-related factors^15^. However, in this study, while RMR was significantly lower among middle-aged adults, no significant differences in LBM and FM were observed between young adults and middle-aged adults. The maintenance of LBM varies between both sexes and shows a considerable variation depending on body training status. Lemmer et al^16^ reported that the intervention of resistance training affected the influence of sex, but no difference was observed in the influence of age. Moreover, chronic aerobic exercise had varied effects in both sexes. In women, chronic aerobic exercise did not affect the age-related decrease of RMR, but affected the age-related decrease in RMR in men^17,18^. Nevertheless, differences in RMR are closely related to the state of energy intake and differ based on usual exercise habits.

Whole-body RMR progressively decreases at a rate of 1-2% per decade with age. This decrease is strongly linked with the reduction in LBM. The reduction in energy expenditure related to aging is the main reason for the decline in whole-body LBM. Another reason for aging induced decrease in energy expenditure is the decrease in the mass and volume of most organs^19,20^. According to Rolfe et al^21^, RMR was determined by other factors such as cellular fractions in tissues, mitochondrial proton leak, and protein turnover. Based on these previous studies, we can observe from our results that middle-aged adults have lower RMR than young adults, even when no difference in body composition was observed among the two groups. Furthermore, although middle-aged adults tend to have higher PA in a week, there is no significant difference in body composition (LBM, FM (kg and %)) between young and middle-aged adults. 

We also observed the younger women had higher body fat percentage (FM %; 30.01 ± 6.06 vs. 28.64 ± 6.28) and blood TG (161.06 ± 72.71 vs. 133.67 ± 65.36 mg/dL) than middle-aged women. Moreover, higher PA (EE per week; 1656.90 ± 1133.34 vs. 2572.46 ± 1156.11 kcal/week) was observed among middle-aged women. In Korea, the rate of obesity and diabetes is consistently increasing among young adults in their 20s. The prevalence of normal weight obesity in normal BMI among adult women over 20 years old is about 30%, it has become a serious issue^22,23^. Therefore, in the present year, Korea started implementing medical checkups for young adults in their 20s. Although our results do not reflect all the Korean young adults in their 20s, we could recognize this phenomenon. Therefore, our results indicate that more PA is required in younger adults. 

From our correlation analysis on body composition, RMR, and PA, we have observed that higher PA level results in decreased FM (%) and blood TG level in young adults. In middle-aged adults, higher PA levels have been observed to result in increased LBM. This is related to increasing RMR in middle-aged adults. Notably, middle-aged adults showed interrelatedness between BMC and PA (EE). For adults aged 18 - 64 years, WHO recommends at least 150 minutes of moderate-intensity PA during the week, at least 75 minutes of vigorous-intensity PA throughout the week, or an equivalent combination of moderate- and vigorous-intensity activity^34^. Our results indicate that, in young aged adults, time spent on moderate-intensity PA was 146.1 ± 18.9 minutes per week and on vigorous-intensity PA was 68.2 ± 37.9 minutes per week. Middle-aged adults were found to spend 391.6 ± 150.7 minutes per week on moderate-intensity PA and 110.0 ± 16.7 minutes per week on vigorous-intensity PA. Young adults were found to be spending lesser time and middle-aged adults, more time, on moderate to vigorous PA, when compared with the WHO recommendation. Many studies suggest that PA (investigated by using doubly labeled water, an accelerometer, and questionnaire) has a strong correlation with body composition and obesity^24-28^. Myers A et al.^29^ demonstrated that moderate-to-vigorous PA is associated with lower adiposity. This study was performed using 71 participants, who were continuously monitored for six to seven days to track free-living PA. This study also tracked actual PA through wearable device armbands. Our study shows similar findings, although our investigation was performed using self-reported PA. 

Several studies^31-32^ have reported that age has a direct effect on RMR due to total body mass (skeletal muscle components) which tends to decrease with aging. Aging reduces LBM and organ mass and is associated with reduction in PA, consequentially reducing RMR. Conversely, our results did not show significant reduction in LBM among middle-aged adults, possibly due to higher levels of moderate to vigorous PA than young adults. LBM is strongly related to BMD^33^ and decrease in BMD increases the risk of bone fracture from osteoporosis. Therefore, more PA is required to prevent obesity and osteoporosis in middle-aged adults and senior adults. Taken together, the results of our study revealed that PA is correlated with the positive effect of reducing body fat mass in young and middle-aged adults. Besides, we confirm that it is important to maintain LBM through PA as age advances. 

The present study has several limitations. Firstly, we calculated PA using GPAQ which included the potential limitation of over-reporting of work, transportation, and leisure time, and so it was difficult to determine the exact type, amount, and intensity of PA. Secondly, we considered a small sample size of middle-aged male participants. However, we could confirm the differences between young and middle-aged adults. Thirdly, to measure RMR, we used indirect calorimetry using a metabolic gas analyzer for only 30 minutes. For a more reliable evaluation in future studies, RMR has to be measured for a longer duration using a human metabolic chamber. 

In conclusion, body composition (LBM and FM) showed no significant difference between young and middle-aged adults, but significant differences are found in RMR and health-related blood variables. However, the PA level tended to higher in middle-aged adults. Moreover, a positive correlation between PA and LBM was observed in middle-aged adults. These results indicate that high PA levels enable LBM and RMR maintenance in middle-aged adults. Therefore, our findings are helpful in understanding the influence of PA on health in Korean adults.
